# What is the relationship between the local population change and cancer incidence in patients with dyslipidemia: Evidence of the impact of local extinction in Korea

**DOI:** 10.1002/cam4.7169

**Published:** 2024-04-10

**Authors:** Wonjeong Jeong, Dong‐Woo Choi, Woorim Kim, Kyu‐Tae Han

**Affiliations:** ^1^ Cancer Knowledge & Information Center National Cancer Control Institute, National Cancer Center Goyang Republic of Korea; ^2^ Cancer Big Data Center National Cancer Control Institute, National Cancer Center Goyang Republic of Korea; ^3^ Division of Cancer Control & Policy National Cancer Control Institute, National Cancer Center Goyang Republic of Korea; ^4^ National Hospice Center National Cancer Control Institute, National Cancer Center Goyang Republic of Korea

**Keywords:** cancer incidence, medical accessibility, population decrease, regional disparity

## Abstract

**Background:**

Changes in the local population are intricately linked to healthcare infrastructure, which subsequently impacts the healthcare sector. A decreasing local population can result in lagging health infrastructure, potentially leading to adverse health outcomes as patients may be at risk of not receiving optimal care and treatment. While some studies have explored the relationship between chronic diseases and local population decline, evidence regarding cancer is insufficient. In this study, we focused on how deteriorating management of chronic diseases such as dyslipidemia could influence the risk of cancer. We investigated the relationship between changes in the local population and cancer incidence among patients with dyslipidemia.

**Methods:**

This cohort study was conducted using claims data. Data from adult patients with dyslipidemia from the National Health Insurance Service–National Sample Cohort conducted between 2002 and 2015 were included. Population changes in each region were obtained from the Korean Statistical Information Service and were used to link each individual's regional code. Cancer risk was the dependent variable, and Cox proportional hazards regression was used to estimate the target associations.

**Results:**

Data from 336,883 patients with dyslipidemia were analyzed. Individuals who resided in areas with a decreasing population had a higher risk of cancer than those living in areas with an increasing population (decrease: hazard ratio (HR) = 1.06, 95% CI = 1.03–1.10; normal: HR = 1.05, 95% CI = 1.02–1.09). Participants living in regions with a low number of hospitals had a higher risk of cancer than those in regions with a higher number of hospitals (HR = 1.20, 95% CI = 1.12–1.29).

**Conclusion:**

Patients in regions where the population has declined are at a higher risk of cancer, highlighting the importance of managing medical problems caused by regional extinction. This could provide evidence for and useful insights into official policies on population decline and cancer risk.

## 1 INTRODUCTION

Korea is currently facing rapid changes in its population structure. The proportion of older adults (aged 65 or older) in Korea was only 5% in 1990 but has been increasing rapidly for decades, and in 2018, it qualified as an “aged society,” with more than 14% of the population being over 65 years old. According to the most recent statistics, in 2022, 17.5% of Korea's population comprised older adults.^1^ This drastic change is because of a decline in fertility rates and an increase in population life expectancy, with the total fertility rate standing at only 0.78 in 2022 compared to 1.48 in 2000.^2^ Besides the age structure, changes in the regional population distribution have also been observed. In 2000, the population of the metropolitan areas (Seoul, Gyeonggi) accounted for 40.9% of the total population but increased to 44.7% by 2022,^3^ consequently leading to a decline in local populations, which is not easily resolved. This issue is one of the core tasks of the current administration and has become a popular topic with a very high public interest.

The decrease in local populations is related to the backwardness of both the environment and infrastructure. Population outflow is accelerating in regions with poor access to large cities or a lack of social resources related to quality of life, such as living welfare, healthcare, living environment, and welfare for people with disability and older adults.^4^, ^5^ It could, therefore, negatively affect the supply of medical resources by decreasing the profitability of medical institutions in local areas. It may also be evaluated as shortages in the elements of accessibility to medical care that can affect the healthcare of local residents, resulting in local patients having longer travel and waiting times for disease treatment, thereby worsening their health management.^6^, ^7^ Previous studies have shown that local extinction of certain aspects of a population could result in negative health outcomes. Some Korean studies have demonstrated a relationship between regional extinction and health performance, including life expectancy, subjective health levels, and the degree to which people suffer from certain diseases.^8^ In addition, regions with a higher risk of extinction are significantly related to amenable mortality.^9^ In the study of chronic diseases, comprehensive management of symptoms, based on primary care, is required, but changes because of local extinction could contribute to worsening health management.^10^

Cancer is the leading cause of death in Korea, and its incidence has steadily increased over the past few decades. For instance, the age‐standardized cancer incidence rose from 402.7 in 1999 to 526.7 in 2021, marking a 30.8% increase.^11^ Given its prevalence, addressing cancer demands comprehensive efforts across prevention, early diagnosis, optimal treatment, and rehabilitation along the care continuum.^11^, ^12^ However, cancer incidence and patterns vary across regions in Korea, as indicated by Koreans.^13^ From 2014 to 2018, regional incidence ranged between 480.5 and 525.9 per 100,000 population based on age‐standardized cancer incidence.^14^ Additionally, according to National Health Insurance data, the number of cancer‐specific code registrations (V193) per 100,000 population for copayment reduction in 2022 varied from 571.9 to 808.3.^15^

While numerous factors can influence cancer incidence, regional disparities may be related to changes in regional populations. Population shifts within a region can impact the functionality of local healthcare programs and the availability of healthcare resources. Disease can exacerbate if appropriate healthcare interventions are not administered during the initial stage of physical abnormalities. Poorly managed dyslipidemia, in particular, can heighten the risk of cancer, and these changes in the local population can also affect well‐managed cases of dyslipidemia.^16^, ^17^ We hypothesized that these demographic changes are relevant to the disparity in regional cancer incidence. Despite the prevalence of chronic diseases such as dyslipidemia or hypertension, few related studies have been conducted. Therefore, this study aimed to explore the relationship between local population decline and cancer incidence among patients diagnosed with dyslipidemia by integrating National Health Insurance data and local population data. It also aimed to provide evidence for policy intervention from a long‐term perspective in Korea.

## 2 METHODS

### 2.1 Data and study participants

Data for this study were obtained from the 2002–2015 National Health Insurance Service–National Sample Cohort (NHIS‐NSC). The NHIS‐NSC data included all medical claims from approximately 2% of the South Korean population using stratified random sampling. As diagnosed chronic diseases such as dyslipidemia are significant risk factors for cancer, this study focused on individuals diagnosed with dyslipidemia (International Classification of Disease, 10th revision [ICD‐10] code: E78) after 2003, with 2002 designated as the washout period. In total, 339,784 individuals aged >20 years were included in this study. Among patients with dyslipidemia, those diagnosed with cancer before the dyslipidemia diagnosis were excluded. Moreover, individuals diagnosed with thyroid, breast, or prostate cancer were excluded from the analysis because of concerns about overdiagnosis in Korea.^18^ Consequently, the final target population comprised 336,883 individuals with dyslipidemia. Regional variables were obtained from the Korean Statistical Information Service (KOSIS) and were used to link each individual's regional code.

All data are available in the Korean National Health Insurance Sharing Service database (https://nhiss.nhis.or.kr) and can be accessed upon reasonable request. The NHIS‐NSC data are secondary and do not contain any identifying information.

### 2.2 Variables

Cancer risk was the dependent variable in this study. Cancer risk was classified based on ICD‐10 codes starting with C. However, thyroid cancer (ICD‐10: C61), breast cancer (ICD‐10: C50), and prostate cancer (ICD‐10: C73) were excluded to mitigate the effects of overdiagnosis attributable to regional characteristics; these cancers have an increased incidence but a lower morality rate.^18^, ^19^, ^20^ Data were analyzed to determine the risk of cancer within or at the end of the follow‐up period (December 31, 2015). The follow‐up period was defined as the date of dyslipidemia diagnosis to the date of cancer diagnosis or December 31, 2015.

Population changes in each region in which individuals were diagnosed with dyslipidemia were analyzed. The population of each region was calculated by dividing the population in 2022 by the population in 2012, using statistics from KOSIS. The data for 2022 were the most recent population data available, and we aimed to analyze changes over a 10‐year period. Region‐related variables were obtained from the KOSIS and used in conjunction with the regional codes of each individual's place of residence, which included the number of hospitals, financial independence, and the aging index. Each variable was measured using the combined regional codes of each individual, and the most recent data from KOSIS were used to calculate the median values for the low and high categories.

Individual characteristics included sex, age, income, region, medical insurance, and the Charlson Comorbidity Index (CCI) score. Cancer was not included in the CCI score because all individuals were diagnosed with cancer. AIDS/HIV was not included because the related data were considered sensitive and were not provided for the sample cohort.

### 2.3 Statistical analysis

The chi‐squared test was used to investigate the general characteristics, which are reported as frequencies and percentages. The incidence rate of cancer risk and 95% confidence interval (CI) were calculated based on a generalized linear model with a Poisson distribution and expressed as the number of patients with cancer per 100,000 person/years. Cox proportional‐hazards regression was used to calculate the association between population changes in each region and the risk of cancer among patients with dyslipidemia, which was determined using the adjusted hazard ratio (HR) and 95% CI. This model accommodates time‐to‐event data, different baseline hazard functions of each disease, and direct comparison of association with outcomes.^21^ The Cox proportional‐hazards regression model specifies that $\lambda \left(t|Z\right)=\lambda_{0}\left(t\right)e^{\beta^{\prime }Z}$, where Z is a vector of covariates of interest, and $\lambda_{0}\left(t\right)$ is a baseline hazard function.^22^, ^23^ Survival time was defined as the number of days from the diagnosis of dyslipidemia (time zero) to the date of cancer diagnosis or December 31, 2015, whichever occurred first. All data analyses were performed using the SAS Enterprise Guide 7.1 (SAS Institute Inc., Cary, NC, USA).

## 3 RESULTS

Table 1 shows the general characteristics of the study population. The regional population was divided into tertiles, whereas the number of hospitals, financial independence, and aging index were divided into medians.

**TABLE 1:** General characteristics of the study population.

Variables	Total		Risk of cancer				
			Yes		No		*p*‐value
	*N*	%	*N*	%	*N*	%	
Total	336,883	100.0	22,629	6.7	314,254	93.3	
Regional‐level characteristics							
Regional population							
Decrease	111,831	33.2	7858	7.0	103,973	93.0	<0.0001
Normal	111,017	33.0	7740	7.0	103,277	93.0	
Increase	114,035	33.9	7031	6.2	107,004	93.8	
Number of hospitals							
Low	166,497	49.4	11,596	7.0	154,901	93.0	<0.0001
High	170,386	50.6	11,033	6.5	159,353	93.5	
Financial independence							
Low	167,067	49.2	11,872	8.0	153,777	92.0	<0.0001
High	172,717	50.8	10,757	7.1	160,477	92.9	
Aging index							
Low	162,490	48.2	10,165	6.3	152,325	93.7	<0.0001
High	174,393	51.8	12,464	7.1	161,929	92.9	
Individual‐level characteristics							
Sex							
Male	162,874	48.3	12,377	7.6	150,497	92.4	<0.0001
Female	174,009	51.7	10,252	5.9	163,757	94.1	
Age							
19–39	61,755	18.3	1331	2.2	60,424	97.8	<0.0001
40–49	74,683	22.2	3328	4.5	71,355	95.5	
50–59	95,917	28.5	6281	6.5	89,636	93.5	
60–69	61,627	18.3	6837	11.1	54,790	88.9	
≥70	42,901	12.7	4852	11.3	38,049	88.7	
Income							
Low	88,107	26.2	5927	6.7	82,180	93.3	<0.0001
Middle	115,724	34.4	7324	6.3	108,400	93.7	
High	133,052	39.5	9378	7.0	123,674	93.0	
Medical insurance							
Insurance (Corporate)	211,690	62.8	13,285	6.3	198,405	93.7	<0.0001
Insurance (Regional)	111,324	33.0	8096	7.3	103,228	92.7	
Medical Aid	13,869	4.1	1248	9.0	12,621	91.0	
Region							
Metropolitan	213,521	62.8	9545	7.1	137,197	92.9	<0.0001
City	112,319	33.1	5434	8.1	80,423	91.9	
Rural	13,944	4.1	7650	9.5	96,634	90.5	
Charlson Comorbidity Index (CCI)							
0	59,134	17.6	881	1.5	58,253	98.5	<0.0001
1	71,025	21.1	2413	3.4	68,612	96.6	
2	63,687	18.9	3565	5.6	60,122	94.4	
≥3	143,037	42.5	15,770	11.0	127,267	89.0	

Table 2 shows the incidence rates of cancer in patients with dyslipidemia. Among 336,883 patients, 22,629 were diagnosed with cancer. The incidence rates for those who lived in areas with decreasing populations, normal population growth, and increasing populations were 272.1 per 100,000 person/years (7858 patients with cancer), 269.8 per 100,000 person/years (7740 patients with cancer), and 237.2 per 100,000 person/years (7031 patients with cancer), respectively.

**TABLE 2:** Incidence rates of cancer risk in patients with dyslipidemia.

Exposure	Number of participants	Number of patients with cancer	Incidence rate (95% CI) per 100,000 person years
Total	336,883	22,629	
Regional population			
Decrease	111,831	7858	272.1 (266.0–278.3)
Normal	111,017	7740	269.8 (263.7–276.0)
Increase	114,035	7031	237.2 (231.6–242.9)

The association between population changes in each region and cancer risk among patients with dyslipidemia is presented in Table 3. Model 1 was adjusted only for regional characteristics, model 2 was adjusted for regional characteristics and socioeconomic status of each individual, and model 3, which was the best‐fitting model in this study based on the lowest log‐likelihood ratio, controlled for all covariates. In model 3, individuals who resided in areas with a decreasing population had a higher risk of cancer compared to those living in areas with an increasing population (decrease: HR = 1.06, 95% CI = 1.03–1.10; normal: HR = 1.05, 95% CI = 1.02–1.09). Participants living in regions with a low number of hospitals had a higher risk of cancer than those in regions with a higher number of hospitals (HR = 1.20, 95% CI = 1.12–1.29). Regions with a high aging index had a higher risk of cancer than those in regions with a low aging index (HR = 1.06, 95% CI = 1.02–1.10).

**TABLE 3:** Association between population changes in each region and cancer risk among patients with dyslipidemia.

Variables	Risk of cancer		
	Model 1	Model 2	Model 3
	HR (95% CI)	HR (95% CI)	HR (95% CI)
*Model fit*			
‐2LL	574254.8	567083.9	561138.2
Regional‐level characteristics			
Regional population			
Decrease	1.09 (1.05–1.13)	1.09 (1.06–1.13)	1.06 (1.03–1.10)
Normal	1.08 (1.05–1.12)	1.08 (1.04–1.11)	1.05 (1.02–1.09)
Increase	1.00	1.00	1.00
Number of hospitals			
Low	1.07 (1.04–1.10)	1.20 (1.12–1.28)	1.20 (1.12–1.29)
High	1.00	1.00	1.00
Financial independence			
Low	1.07 (1.04–1.10)	1.04 (1.01–1.07)	1.01 (0.97–1.04)
High	1.00	1.00	1.00
Aging index			
Low	1.00	1.00	1.00
High	1.11 (1.08–1.14)	1.07 (1.03–1.11)	1.06 (1.02–1.10)
Individual‐level characteristics			
Sex			
Male		1.49 (1.45–1.53)	1.51 (1.47–1.55)
Female		1.00	1.00
Age			
19–39		1.00	1.00
40–49		2.08 (1.95–2.22)	1.78 (1.67–1.90)
50–59		3.22 (3.03–3.42)	2.53 (2.39–2.69)
60–69		5.57 (5.25–5.90)	3.93 (3.70–4.17)
≥70		5.83 (5.48–6.20)	4.02 (3.78–4.28)
Income			
Low		0.96 (0.93–0.99)	0.97 (0.93–1.00)
Middle		0.98 (0.95–1.01)	0.98 (0.95–1.01)
High		1.00	1.00
Medical insurance			
Insurance (corporate)		1.00	1.00
Insurance (regional)		1.15 (1.12–1.18)	1.07 (1.04–1.10)
Medical aid		1.23 (1.15–1.31)	1.07 (1.00–1.14)
Region			
Metropolitan		1.20 (1.10–1.30)	1.23 (1.13–1.33)
City		0.99 (0.94–1.04)	1.02 (0.97–1.07)
Rural		1.00	1.00
Charlson Comorbidity Index (CCI)			
0			1.00
1			2.18 (2.02–2.35)
2			3.46 (3.21–3.72)
≥3			5.90 (5.59–6.41)

## 4 DISCUSSION

The main results of this study suggest that patients with dyslipidemia in regions where the population has declined above the median are at a higher risk of developing cancer. In previous studies, the focus on cancer incidence has primarily been based on the concept of prevention. Studies on regional patterns of cancer incidence in Korea have analyzed the association with preventive aspects of cancer, such as regional factors, eating habits, and coastal accessibility.^24^ Furthermore, based on the concept of medical accessibility, some studies have shown a relationship between regional factors, including healthcare accessibility, such as the number of doctors or hospitals, and disease patterns and outcomes.^25^, ^26^

Based on our findings, this study examined the relationship between changes in regional characteristics and cancer incidence from a different perspective compared to that in previous studies. Previous studies have often associated local population numbers with cancer incidence, suggesting that they reflect access to healthcare and may contribute to overdiagnosis.^27^, ^28^ However, our study reveals a different trend. Even after excluding cancer‐related overdiagnosis, our results demonstrate that the number of patients with cancer increases or decreases because of changes in population size. This implies that alterations in regional population size can significantly impact cancer incidence, regardless of concerns about overdiagnosis. Considering the anticipated overall population decline and the potential disappearance of populations in many cities outside metropolitan areas, our study underscores the importance of devising alternative strategies for local healthcare, particularly in addressing gaps in cancer management arising from changes in the local population dynamics.

In 2022, the Korean government designated nearly 90 regions as “depopulation areas” and created and supported a regional extinction response fund of one trillion won per year. This fund is aimed at actively responding to the crisis of local extinction caused by population decline and is expected to be supported for 10 years from 2022. As the government is focusing on creating solutions in various fields, including the health sector, it is expected that appropriate alternatives to local healthcare will be presented based on the results of this study. Therefore, it is necessary to present appropriate alternatives to solve healthcare issues related to regional extinction based on the evidence from this study.

Our study identified the regional extinction index, which was calculated by assessing the change in the local population numbers to capture population inflow and outflow. This approach provides a comprehensive interpretation of overall population dynamics, diverging from previous studies that focus solely on the economically active population. In previous studies, the regional extinction index was often calculated by dividing the population of fertile women into the elderly population of each region.^29^ The uneven distribution of the population decline crisis across regions is evident, with extinction risk and shrinking cities intensifying in the regional areas of Korea.^30^ Local extinction, where populations vanish from regions, has become a significant national concern. Understanding regional cyclical structures, such as population inflows and outflows, is crucial as regional existence is influenced by the economically active population and by social factors resulting from population movements.^31^

The decline in a local population extends beyond the region simply disappearing; there is also the pressing issue of a lagging medical infrastructure. Smaller population areas often struggle to deliver high‐quality essential medical care because of their limited size and inadequate facility equipment. Hospitals in rural areas face fundamental challenges stemming from conflicts between public interest and profitability. While aspects such as establishing a medical safety net for rural patients and managing emergency medical conditions are crucial for public interest, profitability issues may hinder the maintenance of health and medical institutions.^32^ Additionally, their remote and less accessible locations make it difficult for residents to access healthcare services, significantly impacting the profitability and management of healthcare facilities.^33^ Particularly, rapid advancements in cancer treatment may widen the existing outcome gap among rural patients unless direct efforts are made to understand and address the barriers to high‐quality cancer treatment.^34^ Moreover, underdeveloped healthcare infrastructure resulting from a decline in the local population decline could trigger a vicious cycle of insufficient infrastructure investment.^35^ This could lead to a slowdown in overall economic recovery and further exacerbate the decline in the local population.

According to statistics from the Health Insurance Review and Assessment Service (HIRA), the gap in medical resources has been large by region (average number of doctors per 1000 people = 3.2 in 2022, Seoul = 4.8 [150% of the average]); the gap in medical resources has been worsening (average number of doctors per 1000 people = 2.1 in 2007, Seoul = 2.9 [138% of the average]).^36^ Recently, in Korea, various efforts have been made to reduce medical equity between regions by applying the concept of essential medical care according to the gap in medical resources by region, and friction between the government and stakeholders has been revealed.^37^ A reasonable supply plan for effective regional circulation of medical resources should be created, and health policies that can ensure the sustainability and quality of alternatives should be implemented, as well as a simple quantitative expansion to the close regional gaps. Moreover, in the management of patients with dyslipidemia, one of the chronic diseases, a customized screening and management strategy has to be considered at the regional level, referring to the possibility of local population decline and medical infrastructure leading to severe diseases such as cancer.

It should be noted that the current study has several limitations. First, since the analysis was based on the registered addresses of individuals, there may be some differences between the diagnosed area and actual residence; however, this was the most realistic way to determine the address. Second, the stage or severity of cancer could not be included in this study because cancer severity data are not provided in the NHIS data. Third, this study assumed that regional population changes affect health infrastructure. Conversely, health infrastructure may have a causal relationship with population changes. Therefore, attention should be paid to interpretation. Lastly, other unmeasured factors may have influenced the medical gap among regions, and this study did not account for individual factors, including lifestyle factors such as drinking, smoking, and diet, because of the nature of the claims data.^38^, ^39^ Further research considering these factors will be necessary. Despite these limitations, the strengths of this study are noteworthy. First, this study used national sampling cohort data representing the entire South Korean population to provide useful information on the use of national health insurance and health examinations and create high value‐added policies.^40^ Owing to the representativeness of the data, our results can be generalized not only to South Korea but also to other countries with similar demographic characteristics. Furthermore, this study analyzed data that had been followed up for more than 10 years and is able to analyze population inflow and outflow after a long follow‐up period to identify long‐term relationships and provide useful insights into health policies.

This study identified a significant relationship between local population decline and cancer incidence among patients diagnosed with dyslipidemia. Based on our results, patients with dyslipidemia in regions where the population has declined above the median are at a higher risk of developing cancer. This highlights the importance of presenting an appropriate alternative to local healthcare to address the medical problems caused by regional extinction. Regional extinction caused by population inflows and outflows requires an appropriate solution, as it can cause problems beyond a simple decrease in the population, such as access to medical infrastructure. This study provides useful insights that may guide the official policies for population decline and cancer risk. Further research is required to alleviate the burden of population outflow from the region from a long‐term perspective.

## AUTHOR CONTRIBUTIONS

**Wonjeong Jeong:** Conceptualization (equal); formal analysis (equal); methodology (equal); writing – original draft (equal). **Dong‐Woo Choi:** Methodology (equal); writing – review and editing (equal). **Woorim Kim:** Writing – original draft (equal). **Kyu‐Tae Han:** Conceptualization (equal); supervision (equal); writing – review and editing (equal).

## FUNDING INFORMATION

This paper was supported by the National Cancer Center Grant (2210801‐3). The funding sources did not have interventions such as study design and data interpretation.

## CONFLICT OF INTEREST STATEMENT

The authors do not have any conflict of interests to declare.

## ETHICS STATEMENT

This study was reviewed and approved by the Institutional Review Board of the National Cancer Center (IRB number: NCC2023‐0150), and the requirement to obtain any informed consent was waived by NHIS ethics committee because the data provided by the NHIS were anonymized in compliance with confidentiality guidelines. This study adhered to the principles of the Declaration of Helsinki.

## Data Availability

The datasets analyzed during the current study are available in the NHIS. For obtaining the NHIS National Sampling Cohort, go to the following web site, and submit the application form (https://nhiss.nhis.or.kr). The committee will evaluate that, and notice the determination of deliberation, and then, applicants can use this data after payment of fee. The datasets generated and/or analyzed during the current study are available from the first author on a reasonable request. The data that support the findings of this study are NHIS‐claims data and are stored on a separate server managed by the NHIS.
